# Child Development and Family Human Capital Investment Decisions in Nigeria: a Study of Selected States in the Six Geo-Political Zones

**DOI:** 10.12688/gatesopenres.16379.1

**Published:** 2026-04-14

**Authors:** Mohammed Yelwa, Sarah O. Anyanwu

**Affiliations:** 1 University of Abuja Faculty of Social Sciences, Abuja, Federal Capital Territory, 902101, Nigeria; 2 University of Abuja Faculty of Social Sciences, Abuja, Federal Capital Territory, 902101, Nigeria

**Keywords:** Child Development, Family, Human Capital, Investment Decisions

## Abstract

The study examined child development and family human capital investment decisions in Nigeria. The study focused on household per capita income and family structure using the Nigeria living standard survey for 2018/2019 for the secondary data analysis and a field survey conducted by the researchers in six states in each of the geopolitical zones in Nigeria for the primary data analysis. The study was anchored on household utility maximization theory using the ordinary least squares (OLS) method to analyse the secondary data. Four different results were obtained. First, the result of findings from the OLS estimate revealed that per capita income had no significant impact on Family Human Capital Investment Decisions (FHCID) and male perception of the cost of education had a significant positive impact on FHCID. On the contrary, multi-dimensional poverty index and female perception of the cost of education had an inverse significant impact on FHCID. The second result revealed that average household size, family residence from 1 to 30 minutes proximity to school and 31 minutes and above proximity to school had no significant impact on FHCID. Dependency ratio showed an inverse significant impact on FHCID and family literacy level showed a significant positive impact on FHCID. The third result from the binary logistics regression showed that age, occupation and place of residence of the household head had no significant impact on FHCID. Gender (female-headed household) and education showed an inverse significant impact on FHCID. However, household head years in business or paid employment showed a positive impact on FHCID. The fourth result from the binary logistics regression revealed that marital status had no significant impact on FHCID; family size had a significant negative impact on FHCID; and family structure (type of parents) and number of girl child in the household had a direct impact on FHCID. This study showed complementarities in the home utility function, such that the marginal product of investments rises as family living standards rise. These findings highlight lifetime inequalities and necessitates a special focus on treatments for low-income households. Understanding human capital development and how diverse elements interact is critical to combating poverty and its intergenerational transmission. As a result, this study made several recommendations. First, the importance of persistent action by the government and other donor agencies such as the United Nation Children’s Fund (UNICEF) and The World Bank to address the problems of income inequality and pervasive poverty ravaging Nigeria’s economy. The study strongly recommends that family, especially parents, maintain justice and fairness within the home, to foster constructive, sympathetic and peaceful home, encouraging most children to exhibit excellent academic performance. Third, government agencies and hospitals, especially in rural areas, intensify family planning and birth control campaign to help reduce household size. Fourth, children of the poor be given opportunities for paid employment, to enhance their performance in the school. Fifth, children from poor homes be provided with access to scholarships, free instructional materials and books. Sixth, government and its agencies on education intensify sensitization and campaign for families to embrace Western education, especially in the northern region, promote compulsory primary basic education for all children and prosecute parents of out-of-school children or child labour to serve as a deterrent to others. Finally, the study recommends that non-governmental and religious organizations preach peace and tolerance within the family for the well-being and human capital development of their wards.

## 1. Background to the study

Child development connotes the physical, genetic, psychological and emotional stages that occurs in human beings from birth to adolescence. According to Mensah and Ricart Casadevall (2019) human capital is the knowledge acquired by members of the society, it could be skilled or unskilled and health to enable them realise their potentials as productive members of the society. According to Qamruzzaman et al. (2021), economic growth differs between nations because of the various growth factors in each. Several factors, including investments in human and material resources, foreign direct investment (FDI), financial advancement, inflation, and industrialization, are closely related to growth in all areas. Theories of economic growth have traditionally emphasized the significance of having a strong human resource base and a coherent finance system for sustained prosperity. Sustainable economic growth can be achieved through an effective financial sector and the efficient use of economic resources through the development of human capital ( Abubakar et al., 2015).

Numerous studies conducted over the past three decades have demonstrated that early experiences have a substantial influence on children’s abilities, later development, and, ultimately, adult results ( Almond et al., 2018). Life’s first 5 years provide the groundwork for long-term outcomes ( Currie and Almond, 2011). Due to the young brain’s rapid growth and malleability, investment choices made during this period are critical for building up human capital ( Attanasio et al., 2020). Nevertheless, parental involvement as well as early ability influence later human capital in addition to the biological channel ( Heckman, 2007).

The choice of how much and if at all should be invested in human capital of their children is made by parents, and this choice will have an ongoing effect on each child’s future earnings, possibilities for marriage, and general well-being ( Akresh et al., 2012). Family endowment serves as a proxy for the environment of the family in various ways, including paternal training, compassion, personality, peers, and skill. Unrestricted financial transfers are inadequate at fostering children’s talents, according to recent empirical investigations, and the articles included in this paper corroborate this claim ( Del Boca et al., 2016).

According to Akresh et al. (2012) sibling rivalry has a significant contribution to family human capital investment decision. Sibling rivalry for scarce resources exists inside the family. Having fewer siblings who are comparatively more valuable will benefit a child when market limitations (such as those involving credit, capital, or labour) are present.

In addition to sibling’s rivalry, sibling sex composition counts also. For example, the total investments in sons’ education will be greater if family heads are more charitable toward their male children than their female children, which are frequently the case in most traditional civilizations ( Dammert, 2010). Having an elder sister has a good impact on siblings’ human capital development in terms of education, according to Parish and Willis (1993). Another pattern is older sisters get married early and relieve the family the burden of their welfare. Morduch (2000) and Garg and Morduch (1998) concluded that households with all male siblings had higher levels of completed schooling among early teenagers in Tanzania and Ghana than those with all female siblings. Teenage females spend much time caring for younger siblings at home, which has a negative impact on their academic performance and development of their human capital ( Dammert, 2010).

Based on ground-breaking work by Becker and Tomes (1976), who defined the trade-off between the number of children and their “quality,” several publications have investigated the causes of educational investment inequities for children within a household. Some human capital literature, has discussed how parents distribute certain investments among their children in response to the onset of a child’s human capital endowment. According to economic theory, parental investment patterns might be neutral, compensatory, or reinforcing based on productivity concerns and parents’ dislike of child inequality ( Becker and Tomes, 1976).

The early literature, assumed that investments made at any stage of childhood will have an equivalent impact on the development of adult talents. A scalar measure of cognition (IQ or an achievement test) or “human capital” is the outcome of parental investment in the quality of the child. In the early literature, these ideas are frequently used in conjunction with one another. The technology of skill production and skills themselves are the main topics of current research in the economics of human development. Several of the most fascinating new studies view parent-child, parent-teacher-child, and parent-mentor-child relationships as interactive systems that depend on attachment and scaffolding as key factors in determining a child’s development ( Francesconi and Heckman, 2018).

Recent literature additionally allows for parental time and lack of parental knowledge about children’s skills and effective parenting techniques, taking a more unique view of child investment ( Cunha et al., 2013). It develops and puts into practice an econometric framework that integrates research on how external interventions and home influences affect children’s outcomes ( Cunha et al., 2013). Many see the well-established empirical connection between family income and academic success as evidence of market flaws such as loan limits. The empirical evidence that credit restrictions significantly hamper children’s skill development is not particularly robust, although it is conceptually appealing and open to examination using standard methods ( Heckman and Mosso, 2014). This study investigated the impact of child development on family human capital investment decisions in Nigeria based on topics covered in the evaluated literature.

Inadequate research on family decisions to invest in human capital in Nigeria inspired this study. Numerous studies have been done on the effects of economic growth, structural change, environmental quality, Sustainable Development Goals (SDGs), and (FDI), but little is known about how family human capital investment decisions affect children’s development, particularly in Nigeria. Therefore, the goal of this study was to fill these gaps in the literature. The declining resource endowment of families and the poor economic trends brought on by the COVID-19 virus make this study timely.

### 1.1 Problem statement

Economic growth and economic productivity depend on the development of human capital. Despite this idealistic outlook and government’s programs, such as national directorate of employment (NDE) and industrial training fund (ITF) Nigeria’s human capital development is still at an all-time low, particularly among low-income rural families who lack the means to invest in their children’s futures. The lack of resource endowment in most households is one of the main causes of inadequate human capital development ( Schultz, 2007). The cultural and religious prejudice against girls that permeates most households in the country is another factor that results in insufficient human capital development. Investing in a girl child is a waste of resources since they are considered as a possession that males should own. Others think that girls have no business working in the economy or providing for their families and should only be raised to serve their husbands ( Adekola et al., 2010; UNESCO, 2009).

Another challenge is the size of a household (family size) and its per capita income. Decisions about investing in human capital are also influenced by this challenge although a reasonably large family has little to spend on its children’s human capital development. Sibling rivalry over which child should attend school and which should stay at home to assist with household economic chores like farming and fishing can arise in a relatively small family. This tends to happen more frequently. These issues make it necessary to investigate how family human capital investment decisions affect children’s development in Nigeria.

In light of this, the following research questions were raised as a result of the difficulties covered in the study problem statement: What impact does the family structure have on the human capital development of a child? Do decisions on the development of human capital depend on family per capita income?

### 1.2 Research objectives

The goal of this study is to investigate how the development of children is affected by the family human capital investment decisions in Nigeria. Specifically, to:

i. evaluate the effect of family structure on human capital investment decision of child’s development.
ii. determine the effect of family per capita income on human capital investment decision.

### 1.3 Rationale for the study

Child development is the most important period of life and the issues of child development has been neglected overtime particularly in rural communities in Nigerian. In order to reach their full potential, children need appropriate support from parents in areas of education and healthcare during this period ( Obiweluozor, 2015).

Rossin-Slater et al. (2020), Sheridan et al. (2019), Taguma et al. (2012) have consistently shown that child development will have a direct positive impact on a child’s long-term health outcomes and will improve future opportunities, school attainment and even earning potential. Particularly important is the impact of this period on a child’s emotional and social development, which is vital for their future confidence, communication, relationships, community inclusion, education and mental health.

Many children cannot realize their full potential because of adverse conditions in their environment. For example, evidence from rural northern Nigeria showed that children who receive little sensory stimulation in their rural homes are vulnerable to low-access to education, stunting, low-weight and decreased psychomotor development ( Ezeh et al., 2021). Factors like inadequate nutrition, environmental toxins, unstable caregiving, limited stimulation and stress can all have negative impact on a child’s development. Children living in poverty and fragile living conditions, such as war, insecurity or displacement, are particularly vulnerable to inadequate development. These adverse childhood experiences may also cluster and be compounded over time. For example, a child living in poverty is more likely to experience stress, malnutrition and unstable caregiving, all of which have a negative impact on child development.

Early adverse experiences can have lasting effects on child development, affecting school readiness, learning potential, adult mental and physical health, resilience to stress and conflict resolution, while also straining family resources and perpetuating inter-generational poverty. However, recovery is possible with appropriate intervention and support. Investment in child development policies and programmes will result in long-term returns, as children with adequate support in their early years are healthier, better educated and more likely to contribute to society and the global economy. This was what constituted the basis, rationale and thrust of this study.

## 2. Literature review and theoretical framework

Organizations are emphasizing knowledge supply and scarce resources as a result of the knowledge-based economy to improve organization, competitive advantage, and organizational performance ( Debrulle and Maes, 2014). Knowledge, skills, and abilities are acknowledged as an intangible asset that can be used to sustain an organization. It is crucial to invest in continual training and education and keep up with the rapidly changing trends in global technology to maintain firm competitiveness. Knowledge, skill-building, and training levels of the workforce must be improved in order to maintain a good standard of living ( McConnell et al., 2009).

Economists always advocate for the advancement of human capital as a crucial element of the growth equation, regardless of the status of the economy ( Solow, 1956). According to Lucas (1988) and Barro (1997), the development of human capital has become one of the key components for fostering economic growth through technological innovation and adaptation, reducing inequality, and raising labor productivity as a result of the new growth theories.

The knowledge and skills of the population, which transform it into the workforce as a useful input in the production function, are essential to the productivity of the workforce. However, a competent worker not only increases productivity but also gives economic activity energy. Consequently, a significant component of economic growth is thought to be human capital ( Son, 2010).

The implementation of skill development programs and raising the bar for educational standards are constant, significant investments in human capital development ( Romele, 2013). Investments in human development reduce income inequality in society ( Heckman and Jacobs, 2011) and ensure a higher calibre workforce for the economy ( Deere and Vesovic, 2006), both of which contribute to long-term sustainable economic growth. As a result, human capital development positively influences the development of physical capital in the economy. Developing nations view human capital as a key driver of economic progress ( Lucas, 1990). Numerous empirical studies over the last two decades have looked into the beneficial relationships between the growth of human capital and economic advancement ( Ahsan and Haque, 2017; Chen and Fang, 2017).

With multiplier effects on total productivity, extensive human capital development assures the best use of the available physical and financial resources ( Sulaiman, et al., 2015). Investments in human capital development have long-term benefits since they guarantee a trained workforce. Human capital and technology are the key factors in the real output function. Banerjee and Roy (2014), show that the contribution of human capital can multiply with given efforts as technology advances.

In addition to spending money on human development, some researchers argue that increasing the number of students enrolled in a particular stage of education has a good impact on the nation’s economic development ( Amir et al., 2012). Several studies have shown that increasing educational spending yields higher individual productivity and earnings as well as appreciable social returns. Developing economies will confront difficulties to long-term success without raising enrolment rates and improving education quality ( Qamruzzaman et al., 2021; Hanushek, 2013).

### 2.1 Overview of family and family structure

The concept family has been difficult to define because of the complex its nature. The word ‘family’ means different things to different people and the form and structure of the family has changed overtime, making it difficult to come up with a specific definition that covers the range of existing family structures. To this end, several definitions of the family have been propounded to reflect some of these changes. While some of these definitions are conservative, others are regarded as too broad and sometimes, almost unacceptable. Nevertheless, each definition helps to shed lights on the concept of family. Some of these definitions are cited in the following paragraph.

According to Murdock (1949) defines a family as a social group characterized by common residence, economic cooperation, and reproduction. It comprises adults of both genders, with at least two individuals maintaining an accepted sexual relationship, and one or more children, whether biological or adopted, of the sexually cohabiting adults. Burgess and Lock (1916) describe a family as a collective of individuals bound by marriage, blood, or adoption, living together and fulfilling roles such as husband, wife, parent, sibling, thereby shaping a common culture through interaction. Carrington (1999) portrays family as a network of individuals who express love and care for one another. Weston (1991) defined the family from two perspectives: families of affinity, where individuals without blood ties form family bonds based on shared feelings of belonging, and another based on choice, wherein individuals, regardless of legal or biological relationships, choose to define themselves as a family unit. From these definitions, it is clear that ‘family’, cannot be given one single definition. The family as an institution has different connotations in different places. Therefore, in simple terms, the family can be regarded as a group of people (two or more) usually related by blood (and sometimes not related, perhaps by adoption) living together wherein the adults care for the young. Family is structure on the basis of marriage, residence, descent or ancestry, and on the nature of relations. The influence of family structure on child academic achievement decreased by a quarter to a half when families financial base is weak ( Marks, 2006).

Children who live with two married parents perform better academically, socially, intellectually, and behaviorally than children who reside in other family structures, such as stepparent or single-parent households ( Artis, 2007; Broman and Reckase, 2008). The responsibilities and benefits that define marriage are reflected in these examples. They also reflect the financial and emotional resources that influence some adults to enter stable romantic relationships ( Fomby et al., 2011). Together, tenacity and insurance forms have an impact on child rearing, parent-child relationships, the availability of social services, household organization, and family time usage in ways that are beneficial to children ( Amato, 2010; Foster and Kalil, 2007).

Children’s family structures, regardless of their ages, are only a point on a family structure diagram ( Wu and Martinson, 1993). Cohabitation, divorce, and remarriage all imply that these processes may involve several changes to the status of the family structure. As a result, dynamic family structure measures, comprehension of progress, overall checks of changes, and change categorizations have brought logical capability well beyond static family structure measures to models of child wellbeing and academic achievement. These illustrations span a wide range of instructive ideas, but they are mostly anchored in conduct-related problems. Cavanagh et al. (2006) suggests that they are also quite articulate in their early teens.

### 2.2 Effects of single parent families on children’s academic performance

In single-parent households, there appears to be a decline in affection and nurturing, which in turn impacts the child’s academic performance and overall upbringing ( Mabuza et al., 2014; Falana et al., 2012). Parenting promotes and supports the physical, emotional, social and intellectual development of a child from infancy to adulthood. Parenting refers to the intricacies of raising a child and not exclusively for a biological relationship ( Brooks, 2012). The most common caretaker in parenting is the father or mother or both that is, the child’s biological parent(s) in question. A surrogate may be an older sibling, a step-mother, aunt, uncle, or other family members or a family friend ( Bernstein, 2008). In some cases, government and society may have a role in child-rearing. In many cases, orphaned or abandoned children receive parental care from non-blood relations. Others may be adopted raised in foster care or placed in an orphanage. Parenting skill varies and a parent or surrogate with good parenting skill may refer to as a good parent ( Bernstein, 2008).

In Nigeria the existence of single parent families was formerly unknown and where they existed, were ignored as exceptional cases. Currently, single parenting is the fast-growing family system both inside and outside Nigeria ( Nwachukwu (1998), as cited in Chukwuemeka, 2018). Children are morally upright and emotionally stable when the caring responsibilities are carried out by both parents. The family is the first agent the child first come in contact with and so has a great influence on the child’s physical, mental and moral development. The family lays the foundation of education before the child goes to school and the personality that the child takes to school is determined by the family. The child’s emotional development is traced to his or her home environment. A single parent is one not living with a spouse. The single parent takes most of the responsibilities for raising the child or children. The herculean task of child rearing cannot be done by an individual. As a form of building a family, single parenting is now permissible in our societies which formerly stigmatize such a system because it was acceptable. The family is a big institution and parenting is a supporting and an establishing pillar with societal norms and values being accountable for developing psychological and emotional wellbeing of the child. Single mothers are stigmatized because of patriarchal system of family run in Nigeria ( Falana, Bada and Ayodele, 2012). Culturally, it is unacceptable to live with same-sex parents. A parent leaves remarkable impacts on children’s behaviours, personality and health. For example, a girl cannot share as much with her father as she can with her mother or vice versa ( Falana et al., 2012). Children’s initial point of interaction is with their families, where they observe and learn actions of their caregivers ( Topor et al., 2010). In single parenting, there is only one parent to interact with and emulate. Children learn behaviours from their families and if parents are responsible in some manner the child will learn same ( Bandura, 1965). If parents do not built trustworthy relationships with their children, the probability is high that the children will face difficulties in forming good relationships outside home. However, a positive result could be accomplished only if parents demonstrate the kind of behaviour which they want their children to learn ( Connor and Scott, 2007; Chapman et al., 2004). Attachment is a basic human need to secure relationship between children and caregiver. Bowlby (1958) designed four stages of attachments from infancy as pre-attachment, attachment in making, clear cut attachment, and formation of reciprocal relationship. All these stages build a bond and binds parents and their children emotionally ( Connor and Scott, 2007). Bowlby’s colleague built up another three stages he termed detachment, protest and despair experiences children faced when they were separated from their caregivers/parents ( Chapman et al., 2004). When parents are unable to build stronger relationship in their homes, then there are higher chances that children will face some problems such as psychological disorders, decreased intelligence, increase anger and violent behaviour ( Connor and Scott, 2007; Chapman et al., 2004). However, primary school children are the most fragile because they are still in their formative years, meaning that any disruptions could have everlasting effect on them.

Many studies in Nigeria have focused more on parental involvement in children’s school activities, and little has been done in family structure such as single parenthood and its impact on the child’s academic performance.

### 2.3 Conceptual framework

**Figure 1.**
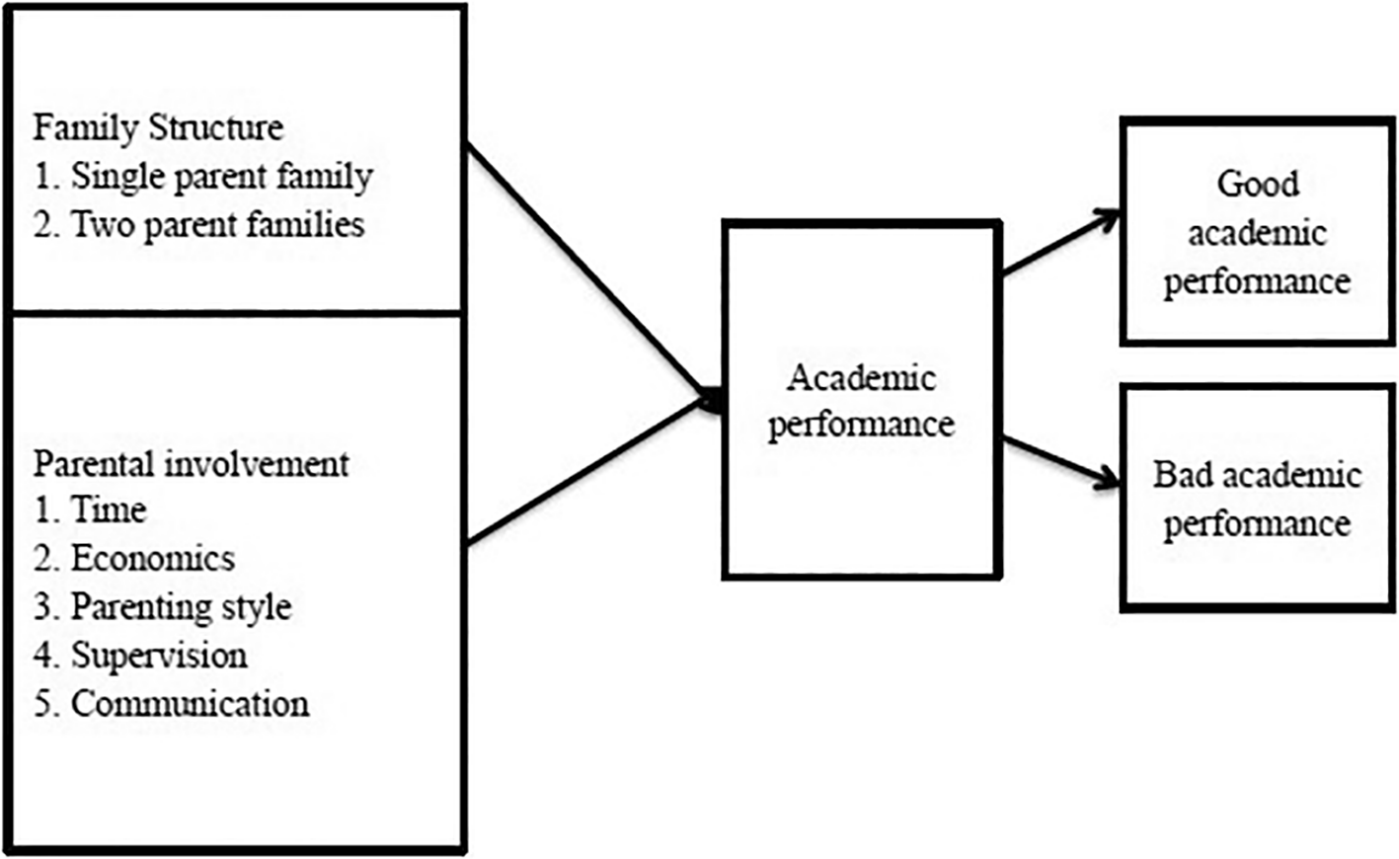
Conceptual framework on how family structure affects children's academic performance. Source: Adapted from Astone and McLahanan (1994).

The conceptual framework identifies a link between parental participation, family structure, and children’s academic performance. Typically, children in a given family structure remain with either their intact parents or a single parent until they turn 18 years old. Regardless of the child’s home arrangement, the diagram suggests that a few key factors can also contribute to a child’s academic achievement. These elements include parental styles, time, supervision, and communication ( Astone and McLahanan, 1991) ( Figure 1).

### 2.4 Theoretical framework

The decision-making process and numerous mechanisms that have an impact the household’s socio-economic choices are identified in early models of household utility maximization. Given their limited resources, households are supposed to maximize their welfare, according to household production models ( Behrman, 1997). Households must decide whether to invest in children’s human capital through resource allocation. This study is anchored on the Separable Earnings Transfer (SET) model.

The (SET) model ( Behrman et al., 1982) makes the assumption that parents are interested in how their children are distributed in terms of wages and transfers, as opposed to just how they are distributed in terms of overall wealth. In this model, the distribution of endowments among children, the desire for equity over productivity, the degree of equal concern across children, and the characteristics of the earnings function all influence parents’ strategy.

The (SET) model suggests that parents may have a variety of choices regarding how their children’s wealth and income are distributed. Therefore, the investment choice could be neutral, compensatory, or encouraging depending on the degree of aversion. For example the model, proposes that parents may opt for a compensating or reinforcing strategy, depending on whether equity or productivity concerns were dominant, if the marginal returns to investment were more significant for children with greater endowments.

## 3. Methodology

The study employed quantitative and qualitative data sources. The empirical analysis used inferential statistics. The inferential statistics employed the Binary Logit regression techniques where the dependent variables were categorical in nature. This paper depended on the works of Francesconi and Heckman (2018). Titled “Symposium on child Development and Parental Investment” they found out that parental time, money and cognitive stimulation significantly impact child development and again that investment in early childhood (0-5 years) have lasting effects on cognitive and non-cognitive skills and finally, they discovered that disparities in parental investment contribute to socioeconomic gaps in child development. Francesconi and Heckmans’s work was builds on the parental investment Model (PIM), which posits that parents decisions about investments in their children affect child development using data from various sources such as National Longitudinal Study of Youth, British Cohort Study, the authors employed econometric techniques such as instrumental variable analysis to estimate the causal effects of parental investment on child development. The data for quantitative were sourced from National Bureau of Statistics (NBS). Data for Nigeria Living Standard Survey 2018/2019 for states excluding Borno were used for the analysis.

The secondary data population comprised all the states in Nigeria and the federal capital territory (FCT) Abuja, excluding Borno State owing to the displacement of its vast numbers of its inhabitants currently residing in internally displaced person (IDP) camps across the country. The population of the primary study comprises the states selected for the study; Ogun (southwest) with a population of 5,217,716; Rivers (South-south) with a population of 7,303,924, Anambra (South-east) with a population of 5, 527809; Nasarawa (North-central) with a population of 2,523,987; Kano (North-west) with a population of 13,076,892 and Bauchi (North-east) with a population of 6,537,314 based on the 2016 population projection figure ( nigerainstat.gov.ng, 2022). The study population cover households from two local governments area (one Municipal, one local) in each of the selected states:

i. Anambra (South-east): Ekwusigo and Awka North
ii. Bauchi (North-east): Ningi and Bauchi Municipal
iii. Kano (North-west): Fagge and Kano Municipal
iv. Nasarawa (North-central): Akwanga and Lafia
v. Ogun (South-west): Ijebu North and Abeokuta North
vi. Rivers (South-south): Port Harcourt and Gokana

The study used the Yamane (1967) sampling technique to determine the sample size. The sampling procedure used was a stratified random sampling technique to select respondents for the study. The respondents were the head of each household from two local government areas (i.e., one municipal and one rural) from the states selected, Anambra, Bauchi, Kano, Nasarawa, Ogun and Rivers. The formula is expressed in equation (3.1)

$$n=\frac{N}{1+Ne^{2}}\;$$ (3.1)

Where:

N: is population size

n: is sample size

1: is constant and;

e: is 0.05 degree of precision or 95% confidence interval

The population for the study was divided in to northern and southern region. The population of the states in the northern region namely Bauchi, Kano and Nasarawa was **22137604** (6537314 + 13076892 + 2523398). Thus, at 95% confidence level, and thus 5% level of precision are assumed for the equation. Therefore, the sample size of the study is determined as follows:

$$\begin{array}{l} n=\frac{22137604}{1+22137604*0.05^{2}}\\ n=399.99≃400. \end{array}$$

Based on the Yamane (1967) formula as presented in Equation (1), a sample of four hundred and one (400) questionnaires was administered. A total of (450) questionnaire were administered to the respondents from the 3 states from the North to compensate for the possibility of non-response. In each of the northern states (Bauchi, Kano and Nasarawa). 150 questionaries’ were administered.

In the southern region, the population of the selected states (Anambra, Ogun and Rivers) is **18049449** (i.e., 5527809 + 5217716 + 7303924)

$$\begin{array}{l} n=\frac{18049449}{1+18049449*0.05^{2}}\\ n=399.99≃400. \end{array}$$

On this basis, (450) questionnaires were administered to the respondents for the 3 states in the south to compensate for the possibility of non-response. A total of 150 questionnaires were administered in each of the southern states, (Anambra, Ogun and Nasarawa). In conclusion a total 900 were sampled for this study.

The case study method was to collect extensive empirical data. The case study design was used to explain the information gathering, formulation, and primary data collection procedures. The unit of analysis comprised selected states from the six geo political zones. The study used 900 questionnaires. For the investigation, the probability sampling method was used. The study specifically used the stratified sampling methodology, which is a type of probability sampling. The stratified sampling technique was used since it allowed for the chance of each instance being selected from the population. Using stratified sampling, it was possible to select respondents from the general population and give them questions. The biggest benefit of using stratified sampling was that it leads to results that were generally accurate and fair. Another benefit is that it ensures that each population subgroup is fairly represented in the sample. The major tool used to collect primary data was a questionnaire. The respondents were given a questionnaire with only closed-ended questions. The respondents who participated in the study chose the appropriate replies from the range of options provided by the closed-ended questions. The study’s goals were accomplished using the questions. Most of the questionnaires were distributed by the researchers during in-person meetings with the respondents. SPSS Statistics 30.0.0 was used to compile the quantitative data gathered and conduct a thorough analysis. The study used inferential statistics for the analysis.

In order to limit the possibility of human bias in the selection of respondents to be included in the sample and to generate a sample that accurately reflected the population under study and allowed for generalizations, purposive sampling was also used in this survey.

### 3.1 Ethical statement

This investigation follows strict ethical standards and concerns with institutional review board (IRB) approval that is, The National Bureau of Statistics (NBS), confirming compliance for the use of Nigeria Living Standard Survey data (NLSS) and administration of questionnaire. Participants were fully informed and gave written consent, with assurances of confidentiality. The study prioritizes participants’ safety and comfort with no conflicts of interest and upholds transparency and impartiality.

### 3.2 Model specification and technique of analysis

The variables used in the secondary data in models 1 and 2 are defined and measured using the National Bureau of Statistics (NBS) Nigeria living standard survey for 2018/2019.

The specified Equation 1: Impact of per capita income on Family Human Capital Investment Decisions

$$\text{lnFHCID}=\beta_{0}+\beta_{1}\mathrm{PCI}+\beta_{2}\mathrm{MPI}+\beta_{3}\mathrm{EMP}+\beta_{4}\text{MPCE}+\beta_{5}\text{FPCE}+\mu \;$$ (1)

Where:

FHCID: is Family Human Capital Investment Decision per state;

PCI: is Per capita income per state (measured by gross national income per capita per state); MPI: is multidimensional Poverty Index per state;

EMP: is total of employed person per state;

MPCE: is male perception of the cost of education per state (percentage population of male who perceived education as being too expensive);

FPCE: is female perception of the cost of education per state (percentage population of female who perceived education as being too expensive);

μ: is stochastic error term;

β _0_: is intercept,

β _1_, β _2_, … , β _5_: is coefficients of the explanatory variables and,

Ln: is natural log

The specified Equation 2: Impact of family structure on Family Human Capital Investment Decisions

$$\text{lnFHCID}=\beta_{0}+\beta_{1}\text{lnAHS}+\beta_{2}\mathrm{DR}+\beta_{3}\mathrm{FLL}+\beta_{4}\mathrm{PTS}1+\beta_{5}\mathrm{PTS}+\mu \;$$ (2)

Where:

FHCID: is family Human Capital Investment Decision per state;

AHS: is average household size per state;

DR: is dependency ratio per state;

FLL: is family literacy level per state;

PTS1: (Proximity to schools) is proximity to school at a distance of zero to 30 minutes;

PTS2: (Proximity to schools) is proximity to school at a distance of zero to 30 minutes;

μ: is stochastic error term;

β _0_: is Intercept;

β _1_, β _2_, … , β _5_: is coefficients of the explanatory variables and;

Ln: is natural log

Based on the nature of this data (cross sectional secondary data), the equations 1 and 2 were estimated using OLS (ordinary least squares) method.

The specified Equation 3: (binary logistic model): Impact of per capita income on family human capital investment decisions

$$\text{FHCID}=\beta_{0}+\beta_{1}\mathrm{AGE}+\beta_{2}\mathrm{EDU}+\beta_{3}\mathrm{OCP}+\beta_{4}\mathrm{YIB}+\beta_{5}\mathrm{POR}+\mu$$ (3)

Where:

FHCID: is family human capital investment decision;

AGE: is age of the household head;

EDU: is education level of the household head;

OCP: is occupation of the household head;

YIB: is number of years the household head in has been in business or paid employment;

POR: is place of residence of the house-head;

μ: is stochastic error term;

β _0_: is intercept

β _1_, β _2_ … β _5_: is a coefficient of the explanatory variables.

The specified Equation 4: (binary logistic model): Impact of family structure on Family human capital investment decisions

$$\text{FHCID}=\beta_{0}+\beta_{1}\mathrm{MS}+\beta_{2}\mathrm{FS}+\beta_{3}\mathrm{FSt}+\beta_{4}\mathrm{HGC}+\mu$$ (4)

Where:

FHCID: is family human capital investment decision per state;

MS: is marital status the household head;

FS: is family size of the household;

FS: is family structure of the household;

NGC: is number of girl children in the household;

μ:is stochastic error term;

β _0_: is Intercept.

β _1_, β _2_ … β _4_: is a coefficient of the explanatory variables.

Inferential statistics were used to assess the study in light of the categorical character of the response variables and study.

## 4. Results and Discussions

Table 1 presents results of the relationship between per capita income and family human capital investment decision using published data sourced (NBS) Nigeria Living Standard Survey 2018/2019 for states excluding Borno. Table 1 captures empirically the effect of per capita income on Family Human Capital Investment Decisions. The independent variables were gross national income per capita (proxy for per capital income), multidimensional poverty index, total number of employed persons, male perception of cost of education and female perception of cost of education. The dependent variable was the log of Family Human Capital Investment Decisions (measured by household expenditure on education).

**Table 1.** Per capita income and family human capital investment decisions.

Dependent variable: lnFHCDID				
Variable	Coefficient	Std. error	t-statistic	Prob.
C	6.71	2.59	2.59	0.01
LNPCI	0.14	0.09	1.45	0.16
MPI	-4.29	0.79	-5.43	0.00
LNEMP	1.20	0.14	8.38	0.00
MPCE	0.01	0.00	2.24	0.03
FPCE	-0.02	0.01	-3.20	0.00
Adj R-sqd = 0.8917	F-stat. = 58.63			

Source: Author’s computation, 2022.

The results in Table 1 revealed that log of per capita income has no significant relationship with the log of Family Human Capital Investment Decisions at 10 % significant level but possesses the potential to increase the log of Family Human Capital Investment Decisions by 13.6 % if the family per capita income increases by 1 %. The multi-dimensional poverty index had a significant negative impact on log of Family Human Capital Investment Decisions such that increase in the multi-dimensional poverty index by a unit will decrease the log of Family Human Capital Investment Decisions by 429 %. In the case of log of total number of employed persons, a significant positive relation exists with Family Human Capital Investment Decisions such that a unit increase in the log of total employed persons will decrease the log of Family Human Capital Investment Decisions by 119.6 %, holding other explanatory variable constant. Male perception of the cost of education was found to have a positive significant impact on log of Family Human Capital Investment Decisions, such that increasing the number of males who consider education too expensive by a unit will increase the log of Family Human Capital Investment Decisions by 1 percent holding constant other explanatory variables. Finally, the female perception of the cost of education shows a significant inverse relationship with the log of Family Human Capital Investment Decisions, such that increasing the number of women who perceived education to be too expensive will reduce the log of Family Human Capital Investment Decisions by 1.8 percent, holding other explanatory variables constant.

The estimated model has adjusted R-squared value of 0.8917 percent, an indication that the estimated model accounts for 89.17 percent of the variation in the dependent variable, this is validated by the significant value of the f-statistic. The diagnostic statistic (Appendix III-V) revealed that the estimated residuals are not serial correlated using the serial correlation LM test. Also, the heteroskedasticity test revealed that the estimated residuals are has a constant variance. Finally, the Ramsey RESET showed that the model is not wrongly specified.

The Table 2 show the relationship between family structure and family human capital investment decision using secondary data sourced from the (NBS) Nigeria Living Standard Survey 2018/2019 for states excluding Borno. The result captures empirically the effect of family structure on Family Human Capital Investment Decisions ( Table 2). The independent variables are log of Average Household size dependency ration, family literacy level (measured by literacy above 12 years), proximity to school 1 (measured by 0-30 minutes distance of family’s residence to school), proximity to school 2 ((measured by 31 minutes and above distance of family’s residence to school). The dependent variable is the log of Family Human Capital Investment Decisions (measured by household expenditure on education).

**Table 2.** Family structure and family human capital investment decisions.

Dependent variable: lnFHCDID				
Variable	Coefficient	Std. Error	t-statistic	Prob.
C	23.60	7.04	3.35	0.00
LNAHS	-1.56	1.04	-1.49	0.15
DR	-0.05	0.02	-2.05	0.05
FLL	0.03	0.01	2.56	0.02
PTS1	0.05	0.14	0.34	0.74
PTS2	0.10	0.14	0.68	0.50
Adj R-sqd = 0.5433	F-stat. = 9.33			

Source: Author’s computation, 2022.

From Table 2, log of average household size showed no significant impact on log of Family Human Capital Investment Decisions at 10 % level of significance but possesses the potential to decrease the log of Family Human Capital Investment Decisions by 155.55 %, holding other variable constant. The dependency ratio showed an inverse significant relationship with log of Family Human Capital Investment Decisions, such that an increase in the dependency ratio by a unit will reduce the log of Family Human Capital Investment Decisions by 4.89 %, holding other independent variable constant. Family literacy level showed a direct significant relationship with the log of Family Human Capital Investment Decisions, such that a 1 % increase in the family literacy level will increase the log of Family Human Capital Investment Decisions by 2.7 %, while holding other explanatory variables constant. Proximity to school in both cases (from 0-30 minutes and 31 minutes and above) had no statistically significant impact on the log of Family Human Capital Investment Decisions.

The estimated model has adjusted R-squared value of 0.5433 %, an indication that the estimated model accounts for 54.33 % of the variation in the dependent variables; this is validated by the significant value of the f-statistic. The diagnostic statistic (Appendices G, H and I) revealed that the estimated residuals are not serial correlated using the serial correlation Lagrange multiplier (LM) test. Further, the heteroskedasticity test revealed that the estimated residuals are has a constant variance. Finally, the Ramsey RESET depicted that the model was not wrongly specified.

**Results of binary logistics regression analysis**

Table 3 addresses the result of field survey and the binary regression results.

**Table 3.** Per capita income and family human capital investment decisions.

Dependent variable: FHCDID					
Variable		Coef (B)	S.E.	P value	Odd Ratio [Exp (B)]
Age				0.12	
	1	-2.298	0.962	0.017	0.1
	2	-1.706	1.673	0.308	0.182
	3	-2.119	2.16	0.327	0.12
Gender		-0.961	0.369	0.009	0.382
Education				0.005	
	1	-1.634	0.424	0	0.195
	2	-23.452	3.39E+03	0.994	0
	3	-23.643	4.04E+03	0.995	0
	4	-23.629	7.41E+03	0.997	0
Occupation				1	
	1	-0.329	4.34E+03	1	0.72
	2	0.152	6.23E+03	1	1.164
	3	0.057	7.05E+03	1	1.059
	4	0.692	6.96E+03	1	1.998
	5	-0.144	0.438	0.742	0.866
	6	-0.302	1.146	0.792	0.739
	7	17.024	4.04E+03	0.997	2.47E+07
Years in service				0.018	
	1	2.497	0.917	0.006	12.141
	2	1.201	1.602	0.453	3.323
	3	3.231	2.092	0.123	25.299
Place of residence	1	-1.037	0.837	0.215	0.354

Source: Author’s computation, from SPSS Statistical package 2022.

A total of 900 questionnaires were administered across the selected states, 773 were returned adequately filled. Of the remaining 127 were poorly filled and others were not returned.

Table 3 presents the result of the binary logit model on the impact of per capita income on family human capital investment decision in Nigeria. The explanatory variables were age, education level, occupation, number of years in business or paid employment and place of residence, and the dependent variable was the Family Human Capital Investment Decisions. Age of the household head is found to have no significant effect on the Family Human Capital Investment Decisions with a probability value (0.120) greater than 0.05 (5 %) level of significance. Gender has an inverse significant impact on the Family Human Capital Investment Decisions with a probability value (0.000) less than the 0.05 level of significance, such that a unit increase in the number of male- headed household that declined from investing in the human capital development of his wards, female- headed household would be less likely to invest in the human capital development of her wards by a factor of 2.617. That is a female head of household would be 2.6 times less likely to invest in the human capital development of her wards than male head of household.

Education level of the household had a significant negative impact on Family Human Capital Investment Decisions with a probability value (0.005) less than 0.05 level of significance. An increase in the number of the household head with a primary school leaving certificate, the less likely it was that the household head with a secondary school certificate would invest in the human capital investment decision of their wards by a factor of 5.128. This implies that household head with a secondary school certificate would be 5.1 times less likely to invest in the human capital development of his wards than a household head with a primary school certificate. The rest of the levels of education had no significant impact on family human capital investment decision.

Occupation of the household head had no significant impact on the family human capital investment decision, since it possesses a probability value (1.00) greater than 0.05 level of significance.

Likewise, place of residence had no significant impact on family human capital investment decision having a probability value (0.215) greater than 0.05 level of significance.

Years in business or paid employment had a significant positive impact on family human capital investment decision with a probability value (0.018) less than 0.05 level of significance. An increase in household head with service years of 1 to 10 years who invested in the human capital development of their wards, the household head with 11 to 20 years in business or paid employment would mostly likely invest in the human capital development of their wards by a factor of 0.082. This implies that household heads with 11 to 20 years working experience would be 0.08 times more likely to invest in the human capital development of their wards than household heads with 1-to-10-year experience. The other years in business or paid employment had no significant impact on Family Human Capital Investment Decisions.

Table 4 presents the result of the binary logit model on the impact of family structure on family human capital investment decision in Nigeria. The explanatory variables are marital status of the household head, family size of the household, family structure of the household and number of girl children in the household. The dependent variable was Family Human Capital Investment Decisions. Marital status of the household head had no significant effect on the Family Human Capital Investment Decisions with a probability value (0.778) greater than 5 % level of significance.

**Table 4.** Family structure and family human capital investment decisions.

Dependent variable: FHCDID					
Variable		Coef (B)	S.E.	P value	Odd Ratio [Exp (B)]
Marital status				0.778	
	1	-0.142	0.434	0.743	0.867
	2	-0.312	0.444	0.482	0.732
Family size				0	
	1	-1.439	0.309	0	0.237
	2	0.169	0.326	0.605	1.184
Family structure				0	
	1	1.078	0.245	0	2.938
	2	0.466	0.355	0.189	1.593
Girl child				0.004	
	1	0.593	0.251	0.018	1.809
	2	1.052	0.45	0.019	2.863
	2	-1.167	0.626	0.062	0.311

Source: Author’s computation, SPSS Statistical package, 2022.

Family size had a significant negative impact on Family Human Capital Investment Decisions; the probability value (0.000) was less than the 0.05 level of significance. Thus, an increase in family size 3 to 6 that declined from investing in the human capital development of their wards, household 7 to 10 would be less likely to invest in the human capital development of their wards by a factor of 4.219. What this means is that a household with family size 7 to 10 would be 4.2 times less likely to invest in the human capital development of their wards than a household with a family size of 3 to 6.

Family structure of household had a significant positive impact on Family Human Capital Investment Decisions, since the probability value (0.000) is less than 0.05 level of significance. An increase in the household with a family structure of two parents, the higher the likelihood that the household with step parents would decide to invest in the human capital investment of their children by a factor of 0.34. This implies that Households with step- parents family structure would be 0.34 times more likely to decide to invest in their children’s human capital development than a household with two parents. Similarly, family structure with single parent would most likely increase the decision to invest in her children human capital investment decision by a factor of 0.628. That is, 0.628 times more likely to invest in her children human capital development than a family structure of two parents.

The number of girl children in the family had a direct impact on Family Human Capital Investment Decisions, with a probability value (0.004) less than 5 % level of significance. An increase in household with 1 to 3 girl children, deciding to invest in their children’s human capital development, the more likely that household with 4 to 6 female children would invest in their children’s human capital development by a factor of 0.55. This means that household with 4 to 6 female children were 0.55 times more likely to invest in their children’s human capital development than to household with 1 to 3 female children. In the same vein, a unit increase in the household with 1 to 3 female children who decided to invest in their children’s human capital development, the more likely than households with 7 or more female children would invest in their children’s human capital development by a factor of 0.35. This implies household with 7 or more female children would 0.35 times more likely invest in their children’s human capital development than to household with 1 to 3 female children. Further, an in increase in households with 1 to 3 girl children, deciding to invest in their children’s human capital development, the more likely than households with no female children would invest in their children’s human capital development by a factor of 3.22. It means that households with no female children would be 3.22 times more likely to invest in their children’s human capital development than to household with 1 to 3 female children.

## 5. Conclusion

The study examined the impact child development on household human capital investment decision. The study focused on household per capita income and family structure using the Nigeria living standard survey for 2018/2019 data for the analysis and fields survey conducted by the researchers in six states in each of the geopolitical zones in Nigeria for the primary data analysis. The research study was anchored on household utility maximization theory using ordinary least squares method to analyse the secondary data, and the primary data were analysed using binary logistics regression. For the secondary data, the exogenous variables of interest were the household head’s age, education level, occupation, the number of years he has worked for a living, and his place of residence. For the primary data, the variables of interest were the head’s marital status, the size of the family, the family structure, and the proportion of girls in the household. Investment decisions made by families in their human capital were considered the endogenous variable.

The study specified four models, two for each data sources. Thus, the study obtained four different results. First, findings from the OLS estimate revealed that per capita income had no significant impact on Family Human Capital Investment Decisions. While male perception on employment and the cost of education had positive significant impact on family human capital development investment decision. On the contrary, multi-dimensional poverty index and female perception of the cost of education had an inverse significant impact on Family Human Capital Investment Decisions.

The second result revealed that average household size, family residence from 1 to 30 minutes proximity to school and 31 minutes and above proximity to school has no significant impact on Family Human Capital Investment Decisions. The dependency ratio showed an inverse significant impact on Family Human Capital Investment Decisions. Family literacy level showed a significant positive impact on Family Human Capital Investment Decisions.

The third result from the binary logistics regression showed that age, occupation and place of residence of the household head had no significant impact on Family Human Capital Investment Decisions. Gender (female headed household) and education showed an inverse significant impact on Family Human Capital Investment Decisions. On the contrary, household head years in business or paid employment showed a positive impact on Family Human Capital Investment Decisions.

The fourth result from the binary logistics regression revealed that marital status had no significant impact on Family Human Capital Investment Decisions. Family size had significant negative impact on Family Human Capital Investment Decisions. Family structure (type of parents) and number of girl child in the household had direct impact on Family Human Capital Investment Decisions.

## 6. Policy recommendations

I. Household size has a negative impact on family human capital investment decision. This is the result of family rivalry emanating from injustice and unfairness of the household and other family members. Therefore, it is strongly recommended that family especially the parents should maintain justice and fairness within the home. By so doing there will be constructive, sympathetic and peaceful homes, ensuring that most children will exhibit excellent academic performance. The study recommends family planning and birth control campaign be intensified by government agencies, non-governmental organizations and hospitals especially in rural areas to help reduce household size.
II. Results showed that poor resource endowment negatively affects child development and human capital investment decision. Therefore, the study recommends that children of the poor be given more opportunities for paid employment. This will enhance their performance in the school. Further, children from poor homes should have access to scholarship, free instructional materials, and books.
III. Literacy level had a significant direct contribution in family human capital investment decision, and most respondents in rural areas were not educated especially in the northern region. The study therefore, recommends that government and its agencies on education should intensify sensitisation and campaign for families to embrace Western education especially in the northern region, promote compulsory primary basic education for all children and prosecute parents of out- of-school children or child labour to serve as a deterrent to others.
IV. The study revealed that family structure (type of parenting) and number of girl children in the family had a direct influence on family human capital investment decisions, such that the children with both parents fare better than children of single parents. Thus, it is recommended that non- governmental and religious organizations need to preach peace and tolerance within the family for the wellbeing and human capital development of their wards.

## Data Availability

The data supporting the findings of this study are publicly available and can be accessed through the following links NIGERIA LIVING STANDARD HOUSEHOLD SURVEY (NLSS) 2018/2019: https://microdata.worldbank.org/index.php/catalog/3827, https://nigerianstat.gov.ng/. Figshare: “APPENDICES”. https://doi.org/10.6084/m9.figshare.28189367 ( Yelwa, 2025). This project contains following extended data:
• APPENDICES - CROSS SECTIONAL DATA (PRIMARY SURVEY) Data are available under the terms of the Creative Commons Attribution 4.0 International license (CC-BY 4.0).
